# Identification of immunogenic proteins of the cysticercoid of *Hymenolepis diminuta*

**DOI:** 10.1186/s13071-017-2519-4

**Published:** 2017-11-21

**Authors:** Anna Sulima, Justyna Bień, Kirsi Savijoki, Anu Näreaho, Rusłan Sałamatin, David Bruce Conn, Daniel Młocicki

**Affiliations:** 1 Department of General Biology and Parasitology, Medical University of Warsaw, Warsaw, Poland; 2 Witold Stefański Institute of Parasitology, Polish Academy of Sciences, Warsaw, Poland; 3 Department of Food and Environmental Sciences, University of Helsinki, Helsinki, Finland; 4 Department of Veterinary Biosciences, University of Helsinki, Helsinki, Finland; 5 Department of Medical Parasitology, National Institute of Public Health - National Institute of Hygiene, Warsaw, Poland; 6 One Health Center, Berry College, Mount Berry, GA USA; 7 Department of Invertebrate Zoology, Museum of Comparative Zoology, Harvard University, Cambridge, MA USA

**Keywords:** *Hymenolepis diminuta*, Immunogenic proteins, Mass spectrometry, Two-dimensional electrophoresis

## Abstract

**Background:**

A wide range of molecules are used by tapeworm metacestodes to establish successful infection in the hostile environment of the host. Reports indicating the proteins in the cestode-host interactions are limited predominantly to taeniids, with no previous data available for non-taeniid species. A non-taeniid, *Hymenolepis diminuta*, represents one of the most important model species in cestode biology and exhibits an exceptional developmental plasticity in its life-cycle, which involves two phylogenetically distant hosts, arthropod and vertebrate.

**Results:**

We identified *H. diminuta* cysticercoid proteins that were recognized by sera of *H. diminuta*-infected rats using two-dimensional gel electrophoresis (2DE), 2D-immunoblotting, and LC-MS/MS mass spectrometry. Proteomic analysis of 42 antigenic spots revealed 70 proteins. The largest number belonged to structural proteins and to the heat-shock protein (HSP) family. These results show a number of the antigenic proteins of the cysticercoid stage, which were present already in the insect host prior to contact with the mammal host. These are the first parasite antigens that the mammal host encounters after the infection, therefore they may represent some of the molecules important in host-parasite interactions at the early stage of infection.

**Conclusions:**

These results could help in understanding how *H. diminuta* and other cestodes adapt to their diverse and complex parasitic life-cycles and show universal molecules used among diverse groups of cestodes to escape the host response to infection.

**Electronic supplementary material:**

The online version of this article (10.1186/s13071-017-2519-4) contains supplementary material, which is available to authorized users.

## Background

Diseases caused by tapeworms are widespread globally, may influence human and animal health, and have a strong economic impact. In South America, Asia, and sub-Saharan Africa, human infections with *Echinococcus* spp. are common [1], whereas infections by *Taenia* spp., *Diphyllobothrium latum* and some of the other cestode species that infect humans are endemic in other regions of world as well [2, 3]. In addition to these highly pathogenic species, there are also cases of human infection with low-pathogenic tapeworms such as *Hymenolepis diminuta*. Human *H. diminuta* hymenolepiasis is a globally widespread zoonotic infection known to be endemic in Asia, southern and eastern Europe, Central and South America, and Africa [4]. Typically, *H. diminuta* parasitizes the small intestine of rodents (mostly mice and rats) but occasionally it infects humans [5, 6]. Most of the reported cases have been documented from children [7–9]. Intermediate hosts for this parasite are beetles, *Tribolium* spp. and *Tenebrio* spp. [6], in which the cysticercoid stage develops. The cysticercoid, as an invasive stage of the tapeworm, enters the rat or human body through the consumption of infected insects, either directly or via contaminated water or food. When it reaches small intestine, it undergoes maturation into the adult stage. As either rat or human may serve as a definitive host for *H. diminuta*, this species represents an interesting model in studies focused on the mechanisms of infection.

Better understanding of a parasite’s adaptations to its way of life and the complexity of interactions between the parasite and the host is possible by introduction of modern techniques of proteomic and genomic research [10–13]. Proteomic studies of helminths have shown new aspects in the parasite-host interrelations involving selected species of tapeworms *Echinococcus granulosus* [14–19]_,_ *Echinococcus multilocularis* [20, 21] and *Taenia solium* [22, 23].

To the best of our knowledge, proteomic studies of cestodes have been conducted exclusively on species developing in mammal host tissues (rats, sheep, humans) under the influence of the host immune system during the period of their differentiation from the hexacanth to metacestode. Metacestodes developing in mammalian hosts (e.g. the taeniids mentioned above) evolved immune-evading strategies when exposed to the presence of host molecules in all of their life-cycle stages, both in the intermediate and definitive host. It is unknown whether similar mechanisms are also present in those cestode species that have invertebrates in their life-cycle. *Hymenolepis diminuta* seems to be a perfect model to explore whether a tapeworm needs to cope with an invertebrate intermediate and the mammalian definitive hosts in different ways [6, 24, 25]. This will help in understanding how *H*. *diminuta* and other cestodes adapt to their diverse and complex parasitic life-cycles.

In this study, we identified *H. diminuta* cysticercoid proteins that were recognized by the sera of *H*. *diminuta*-infected rats. These results show a number of the antigenic proteins of the cysticercoid stage, which were present already in the invertebrate host, without the influence of the mammal host. This study reports on the potential parasite somatic antigens that are encountered by the mammalian host upon infection.

## Methods

### Collection of *Hymenolepis diminuta* cysticercoids

Cysticercoids of *H. diminuta* were isolated from dissected intermediate hosts, *Tenebrio molitor* beetles, 6 weeks after infection, under a dissecting microscope. Cysticercoids were washed 5 times with 100 mM PBS (phosphate buffered saline) to remove debris. Before protein extraction and proteomic analysis cysticercoids were stored at -80 °C.

### Isolation of proteins

After thawing, *H*. *diminuta* cysticercoids were again extensively washed three times in PBS (100 mM) and then mixed with lysis buffer (8 M Urea, 4% CHAPS, 40 mM Tris-base, supplemented with protease inhibitor cocktail; Roche, Berlin, Germany) to solubilize protein components. Then the protein mixture was homogenized in a glass Potter-homogenizer and disintegrated by sonication. The lysis solution was clarified by centrifugation at 14,000× *rpm* for 15 min in an Eppendorf microcentrifuge. Concentration of proteins was measured using the Spectrometer ND-1000 UV/Vis (NanoDrop Technologies, Wilmington, USA). Proteins were kept at -80 °C for further analysis.

### Two-dimensional gel electrophoresis (2DE) and 2DE-immunoblotting

To optimize the conditions for 2DE separation, the first-dimension protein separation was first conducted in the pH range of 3–10 using IPG strips (Bio-Rad, Hercules, USA). We observed that cysticercoid proteins were located in pH 4–7 (results not shown), therefore further separations were performed using IPG strips with pH 4–7 (Bio-Rad, Hercules, USA). The mixture of proteins (approximately 150 μg) were rehydrated overnight in 250 μl of rehydration solution (ReadyPrep™ 2-D Rehydration Buffer, BioRad) and loaded onto a pH 3–10 and pH 4–7 IPG strip for the first-dimension separation. Isoelectric focusing (IEF) was performed using a Protean IEF Cell (Bio-Rad) at 20 °C as follows: 15 min at 250 V, then rapid ramping to 4000 V for 2 h, and 4000 V for 16,000 Vh (using a limit of 50 μA/strip). After IEF, the strips were first equilibrated for 25 min in equilibration buffer (ReadyPrep™ 2-D Starter Kit Equilibration Buffer I, Bio-Rad), followed by a 25 min equilibration in the same buffer supplemented with 2.5% iodoacetamide (ReadyPrep™ 2-D Starter Kit Equilibration Buffer II). The second dimension, SDS-PAGE, was run on 12% polyacrylamide gel in the Midi-Protean Tera Cell (Bio-Rad, USA) with 200 V, for approximately 45 min. All gels were run in the same conditions.

Sera samples were collected four weeks after infection from male Lewis rats, infected with *H. diminuta* at age of about 3 months. Sera samples taken before the infection at day 0 were used as a negative control.

After 2DE, the gels were silver-stained using the Silver Staining Kit according to the manufacturer’s protocol (Krzysztof Kucharczyk Techniki Elektroforetyczne, Warsaw, Poland) or used without staining for 2DE immunoblotting. Further analyses were done using repetitive silver stained gels (> 90% of coverage). Gels were scanned with a GS-800 densitometer (Bio-Rad) and analyzed using Quantity One and PDQuest Analysis Software (Bio-Rad).

For immunoblotting, proteins were transferred by a wet transfer system (Bio-Rad) to a nitrocellulose membrane (Bio-Rad) that was then treated with antisera diluted 1:500 (from experimentally *H*. *diminuta-*infected rats) and then with anti-rat IgG-conjugated to horseradish peroxidase (1:8000, Sigma-Aldrich, Louis, USA). The blots were developed using the SuperSignal West Pico Chemiluminescent Substrate (ThermoFisher Scientific, Waltham, USA) according to the manual, and visualized using GS-800 Densitometer (Bio-Rad) combined with 1-D Analysis Software Quantity 1 (Bio-Rad). The experiment was performed using four biological replicate samples. The imaging of the membrane revealed antigenic spots, of which 42 were selected for mass spectrometry identification.

### LC-MS/MS identification and bioinformatics

Spots excised manually from the silver-stained gels were subjected to standard ‘in-gel digestion’ procedure, in which they were first dried with acetonitrile (ACN) and then subjected to reduction, alkylation, and trypsin digestion (for details see Kordan et al. [26]). Briefly, reduction was done with 10 mM DTT in 100 mM NH_4_HCO_3_ for 30 min at 57 °C. Cysteines were then alkylated with 0.5 M iodoacetamide in 100 mM NH_4_HCO_3_ (45 min in dark at room temperature) and proteins were digested overnight with 10 ng/μl trypsin in 25 mM NH_4_HCO_3_, pH 8.5 (Promega, Madison, WI, USA) at 37 °C. Resulting peptides were extracted in a solution containing 0.1% formic acid and 2% ACN (for details see Kordan et al. [26]).

The tryptic peptides were subjected to liquid chromatography and tandem mass spectrometry (LC-MS/MS) in the Laboratory of Mass Spectrometry, Institute of Biochemistry and Biophysics, Polish Academy of Sciences (Warsaw, Poland). Samples were concentrated and desalted on a RP-C18 pre-column (Waters, Milford, USA), and further peptide separation was achieved on a nano-ultra performance liquid chromatography (UPLC) RP-C18 column (Waters, BEH130 C18 column, 75 μm i.d., 250 mm long) of a nanoACQUITY UPLC system, using a 45-min linear acetonitrile gradient. Column outlet was directly coupled to the Electrospray ionization (ESI) ion source of the Orbitrap Velos type mass spectrometer (Thermo Scientific, Waltham, USA), working in the regime of data dependent MS to MS/MS switch with HCD type peptide fragmentation. An electrospray voltage of 1.5 kV was used. Raw data files were pre-processed with Mascot Distiller software (version 2.5, MatrixScience). The obtained peptide masses and fragmentation spectra were matched with the National Center for Biotechnology Information (NCBI) non-redundant database NCBInr 20160525 (88,005,140 sequences; 32,294,985,422 residues), with a Cestoda filter using the Mascot search engine (Mascot Server v. 2.4.1, MatrixScience). The following search parameters were applied: enzyme specificity was set to trypsin, peptide mass tolerance to ± 20 ppm, and fragment mass tolerance to ± 0.1 Da. The search criteria for the Mascot searches were trypsin digestion with one missed cleavage allowed, carbamidomethyl modification of cysteine as a fixed modification, and oxidation of methionine as a variable modification.

Multidimensional protein Identification Technology - type (MudPIT-type) and/or the highest number of peptide sequences, were selected. The expected value threshold of 0.05 was used for analysis, which means that all peptide identifications had a less than 1 in 20 chance of being a random match. Spectra derived from silver-stained gel pieces usually do not contain enough MS/MS fragmentations to calculate a meaningful FDR, therefore a Mascot score threshold of 30 or above (depending on the value given by Mascot) was used. Classification of the identified proteins was based on Gene Ontology molecular function, biological process, and cellular component information available from the UniProtKB database (http://www.uniprot.org/) and QuickGO (http://www.ebi.ac.uk/QuickGO/).

We used SignalP 4.1 server to predict the presence and location of signal peptide cleavage sites in amino acid sequences in identified proteins of *H. diminuta*. The method incorporates a prediction of cleavage sites and a signal peptide/non-signal peptide prediction based on a combination of several artificial neural networks as described by Nielsen [27].

## Results

### 2DE (two-dimensional gel electophoresis) analysis of cysticercoid proteins of *H. diminuta*

**Fig. 1:**
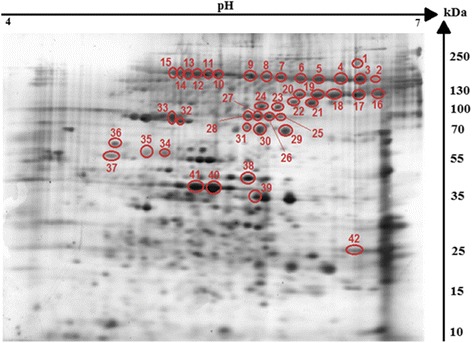
Silver-stained 2-DE protein maps of *Hymenolepis diminuta* cysticercoid protein spots. Cysticercoid proteins were separated on a linear pH range of 4–7 by using IEF in the first dimension and 12% SDS-PAGE in the second dimension. Antigenic protein spots are indicated by red colour

We detected more than 540 protein spots in the proteome of the *H*. *diminuta* cysticercoids, with a pH range of 4–7 and Mw (molecular weight) of 10–250 kDa. Figure 1 represents one of the four replicate silver-stained proteome gels used in further analyses.

**Fig. 2:**
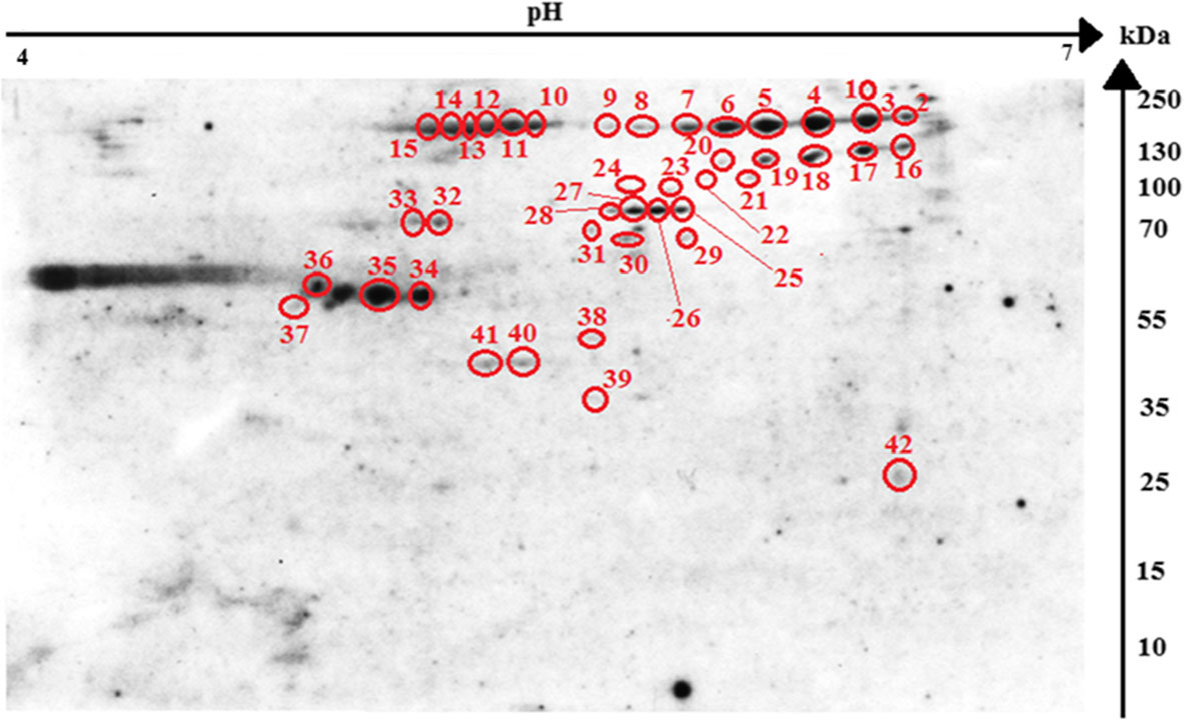
Recognition pattern of *H. diminuta* cysticercoid antigens by antibodies of *H. diminuta*-infected rats. The nitrocellulose membrane shows cysticercoid immunogenic protein spots visualized using SuperSignal West Pico Chemiluminescent Substrate (ThermoFisher Scientific, USA) combined with Quantity One 1-D Analysis Software (Bio-Rad, USA)

2DE-immunoblot (two-dimensional immunoblotting) revealed that 42 spots were positively recognized by the *H. diminuta*-infected rat sera (Fig. 2). All of these spots were successfully identified using LC-MS/MS. As shown in Fig. 2, potentially immunogenic proteins migrated predominantly with a Mw between 55 and 250 kDa. However, a limited number of spots containing antigenic proteins were also observed in the area between 25 and 55 kDa. The proteins were organized in six groups of horizontally adjacent immunorective spots. The first group includes spots labelled 2 to 9 in Fig. 2. The second group of spots is from 10 to 15. Spots 16–20 belong to the third group, whereas 25–28 belong to the fourth. Spots 32 and 33 the fifth group, and 34 to 36 is the sixth.

### LC-MS/MS identification of cysticercoid antigenic proteins *H. diminuta*

The 42 protein spots were subjected to in-gel tryptic digestion and an equal amount of peptides was subjected to LC-MS/MS analysis. We identified 70 potentially antigenic proteins (Table 1). As no whole-genome/proteome database is yet available for *H. diminuta*, protein identification in the present study was conducted against protein sequences available for other cestode species. Several proteins were identified from multiple spots (Additional file 1: Table S1) with differing pI and MW values, suggesting that the proteins in question have undergone post-translational modification (proteolysis or charged modification). Most of the analyzed spots contained more than one protein, e.g. a relatively high number of proteins were identified from spots number 30 and 32 (10 proteins), 35 (11 proteins), 38 (17 proteins), 39 (16 proteins) and 41 (13 proteins). However, most spots taken into consideration contained less than 3–4 proteins. Proteins that appeared to be most frequently identified from multiple spots are: actin cytoplasmic 2 (14 spots), hypothetical transcript (12 spots), procollagen-lysine 2-oxoglutarate 5-dioxygenase (8 spots), and type II collagen B (16 spots) (Table 1). We also noticed the presence of individual proteins identified only in selected spots (Table 1). Most identified proteins are structural and heat-shock family proteins, and selected potentially antigenic proteins are enzymes e.g. belonging to the cysteine proteases family (Table 1, Additional file 1: Table S1). The highly cross-reactive protein spots matched type II collagen B, hypothetical transcript and collagen alpha-1 (XXVII) chain, actin, myosin heavy chain, procollagen-lysine 2-oxoglutarate 5-dioxygenase, HSP3 family member, and tubulin alpha-1C chain (Table 1, Additional file 1: Table S1). The molecular weight of the aforementioned protein spots ranged between 55 and 250 kDa. The spots 37–42 (Fig. 2) with MWs ranging from 25 to 55 kDa and pH from 5 to 6, we found to contain 5 proteins, such as tubulin beta-2C chain, HSP 70, small HSP, actin, and major egg antigen.

**Table 1:** Alphabetical list of identified cysticercoid antigenic proteins with spot numbers and recognition of potentially signaling/secretory proteins

Protein name	Spot number (Number of spots)	Signal protein^a^
26S protease regulatory subunit 6A (*Echinococcus granulosus*)	38, 39 (2)	NO
26S protease regulatory subunit 7 (*Echinococcus granulosus*)	38 (1)	NO
78 kDa glucose-regulated protein	32, 38 (2)	YES
Actin, cytoplasmic 2 (*Echinococcus granulosus*)	8, 10, 11, 20, 24, 27, 29, 30, 31, 35, 38, 39, 40, 41 (14)	NO
Actin, partial (*Diphyllobothrium dendriticum*)	2, 11, 37, 42 (4)	NO
Actin-1	40, 41 (2)	NO
Actin-2	10, 39, 40, 41 (4)	NO
Actin-5	40, 41 (2)	NO
Actin-6	2, 10, 26, 31 (4)	NO
Annexin A7 (*Echinococcus granulosus*)	42 (1)	NO
Apolipoprotein A I binding protein (*Hymenolepis microstoma*)	36 (1)	NO
ATP dependent RNA helicase Ddx1 (*Hymenolepis microstoma*)	22 (1)	NO
ATP dependent RNA helicase DDX31 (*Hymenolepis microstoma*)	38, 41 (2)	NO
Beta tubulin (*Hymenolepis microstoma*)	37, 38, 41, 37 (4)	NO
Beta-tubulin isoform 2, partial (*Echinococcus granulosus*)	37 (1)	NO
Calpain A (*Hymenolepis microstoma*)	30, 34, 35, 36, 37 (5)	NO
Chaperonin containing TCP1 subunit 2 (beta) (*Hymenolepis microstoma*)	39 (1)	NO
Chaperonin containing TCP1 subunit 5 (epsilon) (*Hymenolepis microstoma*)	39 (1)	NO
Collagen alpha 1(V) chain (*Hymenolepis microstoma*)	9, 17, 15 (3)	NO
Collagen alpha 2(I) chain (*Hymenolepis microstoma*)	15 (1)	YES
Collagen alpha-1(XXVII) chain (*Echinococcus granulosus*)	1, 6, 7, 23 (4)	YES
Collagen type i ii iii v xi alpha (*Echinococcus granulosus*)	10, 12, 13, 15 (4)	NO
Dihydrolipoyllysine residue succinyltransferase (*Hymenolepis microstoma*)	38 (1)	NO
Dihydropyrimidinase 2 (*Hymenolepis microstoma*)	35 (1)	NO
Elongation factor 1-a, partial (*Hymenolepis diminuta*)	38 (1)	NO
Filamin (*Hymenolepis microstoma*)	30, 32, 33, 39, 39 (5)	NO
Glucose regulated protein GRP78 (*Spirometra erinaceieuropaei*)	32, 33, 42 (3)	YES
Gynecophoral canal protein (*Hymenolepis microstoma*)	26, 27 (2)	YES
Heat shock 70 kDa protein, partial (*Mesocestoides corti*)	29, 30, 38, 41 (4)	NO
Heat shock cognate 70 kDa protein	42 (1)	NO
Heat shock cognate protein (*Echinococcus granulosus*)	41 (1)	NO
Heat shock protein 60 (*Echinococcus multilocularis*)	34 (1)	NO
Heat shock protein 70 (*Hymenolepis microstoma*)	29, 30 (2)	NO
Heat Shock protein family member (hsp 3) (*Hymenolepis microstoma*)	32, 33(2)	YES
Hypothetical transcript (*Hymenolepis microstoma*)	3, 4, 5, 30, 31, 34, 35, 38, 39, 40, 41, 42 (12)	NO
Lamin dm0 (*Hymenolepis microstoma*)	38 (1)	NO
Lysosomal alpha glucosidase (*Hymenolepis microstoma*)	25 (1)	YES
Major egg antigen (p40) (*Hymenolepis microstoma*)	39 (1)	NO
Mitochondrial ATP synthase (*Spirometra erinaceieuropaei*)	41 (1)	NO
Mitochondrial processing peptidase beta subunit (*Hymenolepis microstoma*)	38 (1)	NO
Myosin heavy chain (*Hymenolepis microstoma*)	12, 32, 33, 34, 35 (5)	NO
Myosin heavy chain non muscle (*Hymenolepis microstoma*)	14, 32, 33, 34 (4)	NO
Myosin heavy chain, striated muscle (*Echinococcus granulosus*)	12, 32, 35 (3)	NO
Myosin-11 (*Echinococcus granulosus*)	32, 33, 34, 35 (4)	NO
NADP dependent malic enzyme (*Hymenolepis microstoma*)	38 (1)	NO
Nuclear pore complex protein Nup205 (*Hymenolepis microstoma*)	29 (1)	NO
Paramyosin (*Hymenolepis microstoma*)	32 (1)	NO
Phosphoenolpyruvate carboxykinase (*Hymenolepis microstoma*)	29, 30, 36 (3)	NO
Procollagen lysine 2-oxoglutarate 5-dioxygenase (*Hymenolepis microstoma*)	24, 25, 26, 27, 28, 29, 30, 31 (8)	YES
Putative cyclin-H (*Echinococcus granulosus*)	42 (1)	NO
Putative HSP20 related protein (*Echinococcus multilocularis*)	39 (1)	NO
Radixin (*Hymenolepis microstoma*)	36 (1)	NO
Retinoblastoma binding protein 4 (*Hymenolepis microstoma*)	37 (1)	NO
Small heat-shock protein (*Taenia solium*)	39 (1)	NO
Spectrin alpha actinin (*Hymenolepis microstoma*)	34, 35 (2)	NO
Spectrin alpha chain (*Echinococcus granulosus*)	32, 33, 41 (3)	NO
Spectrin beta chain (*Hymenolepis microstoma*)	27 (1)	NO
Stomatin protein 2 (*Hymenolepis microstoma*)	39 (1)	NO
Stress-70 protein (*Echinococcus granulosus*)	29, 30 (2)	NO
Succinate coenzyme A ligase, GDP forming, beta subunit (*Hymenolepis microstoma*)	38 (1)	NO
Succinyl-CoA ligase (GDP-forming) subunit beta (*Echinococcus granulosus*)	38 (1)	NO
Talin-1 (*Echinococcus granulosus*)	36 (1)	NO
Transforming growth factor-beta-induced protein ig-h3 (*Echinococcus granulosus*)	25, 26, 27 (3)	YES
Tubulin (*Spirometra erinaceieuropaei*)	35 (1)	NO
Tubulin alpha-1C chain (*Echinococcus granulosus*)	34, 35 (2)	NO
Tubulin beta 1 chain (*Hymenolepis microstoma*)	37, 39, 40, 41 (4)	NO
Tubulin beta 2C chain (*Hymenolepis microstoma*)	37, 38, 39 (3)	NO
Tubulin beta-2 chain	38, 40, 42 (3)	NO
Tubulin beta-3 chain	39 (1)	NO
Type II collagen B (*Echinococcus multiloculari*s)	1, 2, 4, 6, 8, 16, 17, 18, 19, 20, 21, 22, 23, 24, 33, 35 (16)	NO

^a^The presence of secretory/signal proteins predicted with the use of SignalP 4.1 Server software; YES, potentially secretory protein; NO, negative search result

### Gene ontology (GO) of the potentially antigenic proteins of *H. diminuta* cysticercoid

**Fig. 3:**
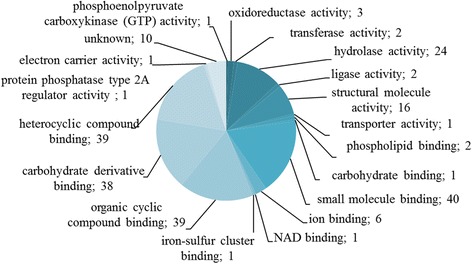
Identified cysticercoid antigenic proteins categorized by their molecular functions according to gene ontology (GO) information obtained from UniProtKB and QuickGO databases

**Fig. 4:**
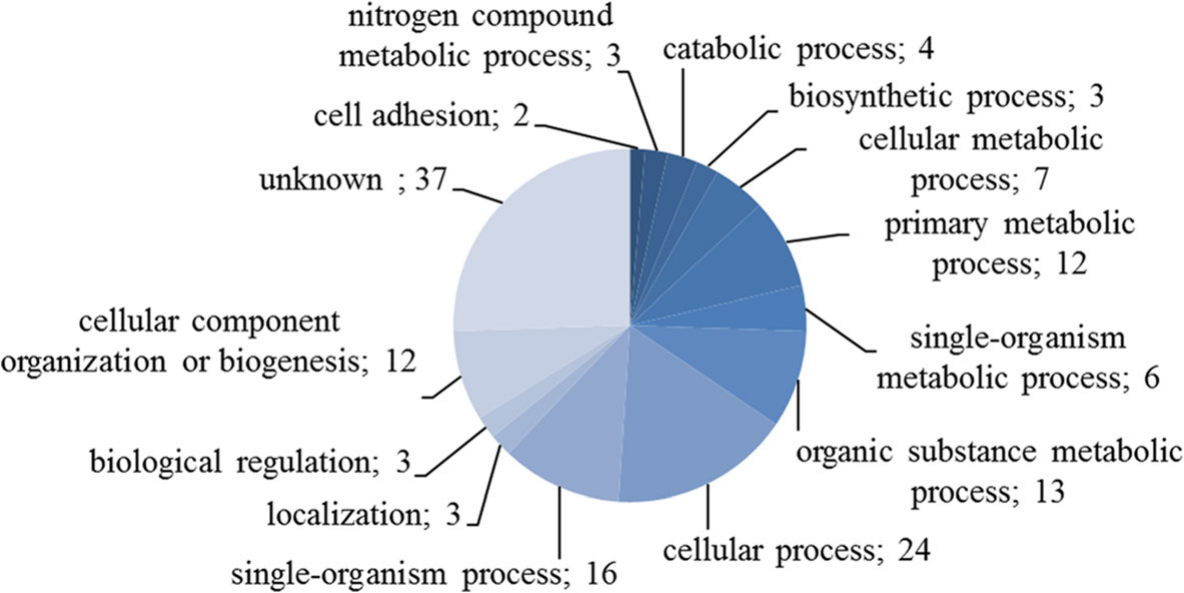
Identified cysticercoid antigenic proteins categorized by their biological processes according to gene ontology (GO) information obtained from UniProtKB and QuickGO databases

**Fig. 5:**
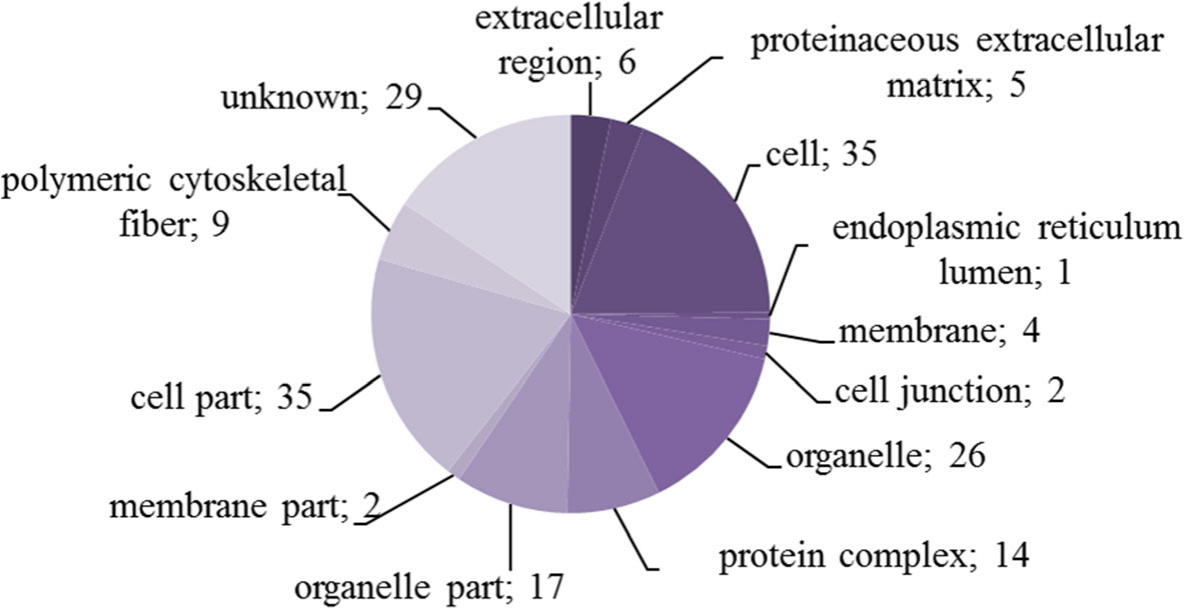
Identified cysticercoid antigenic proteins categorized by their cellular component category according to gene ontology (GO) information obtained from UniProtKB and QuickGO databases

Antigenic proteins identified were grouped according to molecular function (60 proteins), cellular component (41 proteins), and biological process (33 proteins) (Figs. 3, 4 and 5).

Eighteen subcategories were assigned to molecular functions (Additional file 1: Table S2). Most of the assigned molecular functions were associated with: small molecule binding (GO:0036094; 40), organic cyclic compound binding (GO:0097159; 39), carbohydrate derivative binding (GO:0097367; 38), and heterocyclic compound binding (GO:1901363; 39). Altogether 10 cysticercoid proteins have unknown molecular functions.

Biological processes were associated with thirteen subcategories. A large number of potentially antigenic proteins were related to primary metabolic processes (GO:0044238; 12), organic substance metabolic processes (GO:0071704; 13), cellular processes (GO:0009987; 24), single-organism processes (GO:0044699; 16), and cellular component organization or biogenesis (GO:0071840; 12). Among 70 identified proteins, 37 had no established/described biological process (Additional file 1: Table S2).

Cellular components were classified into twelve subcategories (Additional file 1: Table S2). Most of the proteins were classified to cell (GO:0005623; 35), cell parts (GO:0044464; 35), and organelles (GO:0043226; 26). For 29 of the 70 cysticercoid proteins, we were unable to establish an associated cellular component.

With the use of SignalP 4.1 server we were able to predict which of the identified *H. diminuta* proteins are potentially secretory/signal molecules. We predicted the presence of nine potentially secretory/signal proteins. The results of this analysis are shown in Table 1.

## Discussion

Altogether 70 potentially antigenic proteins were identified in this study via a combination of classical 2DE and immunoblot techniques. Our results show that among cysticercoid proteins of *H*. *diminuta*, there are also proteins previously noticed as antigens or proteins being involved in mechanisms of host immune evasion and/or immune modulation in other helminths such as: actin, calpain, HSP70 and HSP60, major egg antigen, myosin, paramyosin [14, 19, 21, 28, 29]; however, we also pointed out some molecules with roles in parasite-host crosstalk that have never been considered in cestodes (e.g. procollagen, collagens, RBBP). Recent data proved that adult *H. diminuta* tapeworms effectively modulate the host immune system [30–32]; however, nothing is known about immunomodulatory and antigenic properties of its metacestodes.

Since our study focuses on the somatic proteins of the cysticercoid taken directly from the intermediate host, future experiments will include experimental infection of rats and proteome and secretome analysis of the juvenile *H. diminuta* tapeworms taken directly form rat intestines soon after establishment. This would provide us with the whole view on the mechanisms involved during the early infection.

Among the proteins identified by us, most are components of the cytoskeleton or muscle system, including extracellular matrices; however, we also noticed molecules involved in metabolic processes, e.g. detoxification. These categories include the majority of proteins already described as modulators of the host-parasite relationships in helminths, as those are preferentially ‘seen’ by the host during infection. Several proteins classified as structural or metabolic are known to play key roles during the process of invasion [22]. Cytoskeleton proteins were highly expressed in cysticercoids of *H. diminuta*. The presence of actin, tubulin, myosin, and paramyosin has been described in the metacestodes of *E. granulosus* causing hydatid disease, and in adult tapeworms as exhibiting antigenic properties [14, 33]. Upregulation of cytoskeleton proteins may be characteristic for the metacestode juvenile worms [34]. Based on our data, we suggest that the higher expression of cytoskeleton proteins in juvenile worms may indicate their role in the active motility of cestodes and in the morphological change from metacestodes and pre-strobilated juveniles to adults, especially in the formation of proglottids. It is possible that upregulation in the expression of cytoskeletal proteins occurs as a consequence of intensive cell proliferation during the differentiation of the *H. diminuta* cysticercoid into the adult parasite and as a response to host immunity. The increased expression levels and the rapid growth of the parasite may expose cytoskeletal proteins to the host immune system and therefore structural proteins of *H. diminuta* were found to be immunogenic [35]. The same could be true for alpha- and beta-tubulins, which are components of microtubules involved in cell division, motility, and polarity. Beta-tubulin is also a target for commonly used benzimidazole anthelmintics [36]. Simultaneous presence of both structural and stress-related proteins in *H. diminuta* may be associated with complex and constant influence of the host immune system and damage repair. The balanced interplay between structural and stress molecules is probably one of the survival factors adapted by parasites during coevolution with their hosts. Damage repair might explain the high number of structural and stress-related proteins observed in cestode immature stages, including cysticercoids of *H. diminuta*.

Paramyosin is one of the interesting proteins identified in cysticercoids of *H. diminuta*. It is very immunogenic and has been proposed to protect invading helminths from immune attack by ‘decoy’ binding proteins of the complement pathway [22]. Paramyosin was identified at the helminths’ surface or in their secretome, and is believed to represent multifunctional modulators of the host immune response [15].

Cytoskeletal and heat-shock proteins (HSPs) were identified as immunodominant among the identified antigens in protoscoleces of *E*. *multilocularis* [21]. Immunoblotting has demonstrated that cytoskeletal and HSP proteins are also present in the secretome of adult *H*. *diminuta* [35]. Indeed, our results show that in *H*. *diminuta* cysticercoids, heat-shock proteins (HSP60, HSP70, HSP20, HSP3, sHSP) are represented as one of the dominant protein families. The HSP family has been previously studied as a potential vaccine candidate [37] similar to calpain, another important protein considered as a vaccine candidate [33, 38], which was also present in several immunoreactive spots of *H. diminuta* cysticercoids.

In *Schistosoma japonicum*, HSP70 was shown to induce an early humoral immune response and may be a good target for immunodiagnosis [39], whereas in adult *Trichinella spiralis* HSP70 underscores its potential as a vaccine candidate [40]. As HSP70 is believed to play an important role in infection, its presence in the *H*. *diminuta* cysticercoid stage may be associated with immunomodulatory activity resulting in successful invasion and survival. Another protein belonging to the HSP family, which was identified from one of the immunoreactive spots of *H*. *diminuta* cysticercoids, is HSP60. The most recent study on *S. japonicum* [41] identified egg-derived HSP60 as a major parasite contributor of regulatory T-cell (Treg) induction among egg antigens [42]. Ben Nouir et al. [43] used monoclonal IgM antibody specific for *Strongyloides ratti* HSP60 and revealed that vaccination with HSP60 conferred protection to infection in a murine model. In relation to our results, the presence of HSP60 among identified proteins may suggest that *H*. *diminuta* promotes molecular mechanisms that induce Treg cells.

Small-HSPs (sHSPs) are considered to be a crucial research focus in the fight against parasitic diseases [44]. sHSPs can induce an immune response in the host, thereby generating potential protection against the disease [44]. However, the information available about their role is still insufficient. The presence of sHSPs in *H*. *diminuta* cysticercoids makes this parasite a possible model to be used in future studies on the role of sHSPs in host-parasite systems.

A group of proteins recognized in *H. diminuta* cysticercoids belongs to collagens. In general, collagens have been classified as proteins carrying antigenic and immunogenic properties [45, 46]. To the best of our knowledge, this is the first study to show potential immunogenicity of collagens forming the metacestode cyst (collagen alpha, procollagen lysine, and collagen B type II). Most probably the collagens detected in metacestodes of *H. diminuta* constitute the cyst wall of cysticercoids. Lee et al. [47] have described the presence of collagen in the wall of cysts formed by *Cysticercus fasciolaris* (metacestode of *Taenia taeniaeformis*) in different organs of wild rats. These authors showed that various types of collagen are engaged in constructing the cysts at different stages. Similarly, collagen was observed in a layer surrounding *Taenia solium* cysticerci in swine [48]. Mixed types of collagens have also been observed to be moderately distributed within the inflammatory infiltration surrounding cysticerci of the parasite *Taenia crassiceps* [49]. Taken together, these studies suggest that high immunogenicity of collagens may help to establish the infection.

Apart from structural proteins, molecules engaged in metabolic processes such as phosphoenolpyruvate carboxykinase (PEPCK) were also identified in this study. Protein identification of hydatid cysts and adults of *E. granulosus* revealed the dominance of paramyosin, actin, and PEPCK in the adult tapeworms. PEPCK proteins are directly involved in a variety of pathways, including the excretory, endocrine, and carbohydrate-metabolism pathways [33].

Other metabolic proteins of the cysticercoid include 78 kDa glucose-regulated protein (GRP-78) and apolipoprotein A-I (ApoA-I). GRP78 is an immunoglobulin member of the HSP70 protein family [50]. It has been found that this protein is an immunodominant antigen in echinococcal disease [51]. The results of research by Yun et al. [52] suggest that GRP78 functions as a molecular chaperone in adapting parasites to the new host environment. Whereas apolipoprotein A-I is the major apolipoprotein of high density lipoproteins (HDL) and has an important role in the regulation of the lipid transport, stability, structure, and metabolism of HDL particles [53]. In addition, ApoA-I was identified as a biomarker of *T. solium* cysticercosis [54]. Our research shows that ApoAI is a potential antigenic protein recognized by antibodies of infected animals; however, Bernthaler et al. [55] found that EmABP (*E. multilocularis* apolipoprotein) does not work as a parasite antigen during active infection.

Interestingly, one of the immunogenic proteins characteristic for *H. diminuta* cyticercoids is the major egg antigen p40 (mp40). In *S. mansoni*, the major egg antigen p40 (Smp40) is well characterized as an immunogenic protein [56, 57], not only in animal models, as Smp40 has been described as immunogenic in humans [58]. In addition, Abouel-Nour et al. [57] indicated the usefulness of Smp40 as an anti-pathology schistosomal vaccine candidate by decreasing fibrosis and inhibiting granuloma formation. On the other hand, *S. japonicum* egg antigen p40 (Smj40) can be used as a marker for early diagnosis of schistosomiasis [59]. As such, the potential of mp40 in prevention of diseases caused by tapeworms requires more research.

It has been demonstrated that chronic invasion caused by parasites may become a significant carcinogenesis factor and induce cancer development in host tissues [60]. Recent evidence from one human case indicates that infection with the low-pathogenic tapeworm *H. nana*, a close relative of *H. diminuta*, may cause a life-threating situation due to invasion of human tissue by genetically altered tapeworm cells [61]. *H. diminuta* is a common laboratory model of *H. nana* infections, therefore information clarifying its interactions with the host may also provide useful details concerning development of parasite-originated malignancies in humans.

We found the presence of retinoblastoma binding protein (RBBP) in the cysticercoid of *H. diminuta*. Gene *pRBBP* is one of the most extensively studied tumour-suppressor genes [62]. Since RBBP represents an important tumor suppressor protein, its dysfunction is a key in several human tumors. Moreover, molecular and biological functions of proteins belonging to the RBBP family, such as RBBP6, are associated with carcinogenesis in humans [63]. Therefore, mutations in RBBP genes may result in malignant transformations in metacestode cells. Interestingly, malignant transformations have never been observed in adult cestodes. The efficacy of anthelminthic drugs (e.g. albendazole) against clonal proliferations of tapeworm stem cells is questionable or even ineffective as observed in *H. nana* [61]. Thus, other tumor proteins noted in cysticercoids and other metacestodes may be considered as potential candidates for anti-invasive helminth cellular proliferation therapies, and diseases caused by metacestodes in general. This may include cysticerci and hydatid cysts of taeniids. However, additional data on the presence and expression level of *RBBP* genes is essential to assess the potential use of anti-RBP therapies in cestode-malignant transformations in human host tissues.

## Conclusions

To our knowledge, the present study represents the first identification of the antigenic proteins of the metacestode form of a non-taeniid cestode *H. diminuta*. Many of the immunogenic proteins recognized, are known to be associated with the immunomodulation of the host in response to infection. Cysticercoids of *H. diminuta* are armed with mechanisms known from other parasitic helminths invading tissues of mammals, and the identified proteins are known to play crucial roles in host-parasite interactions. The results suggest that the similarities observed in the morphology and composition of the neodermatan tegument (myosins, actins, radixin, spectrin, tubulins, filamins) provoke an immune response of the host. In response to the host’s reaction, *H. diminuta* cysticercoids evolved immunomodulatory molecules (paramyosin, HSPs, sHSPs, GRP78), which are considered to be engaged in preventing parasite expulsion from the host and guaranteeing successful invasion and long-term survival. However, to clarify the roles of the individual proteins in host-parasite interactions, mechanisms of invasion, and survival strategies, additional experiments and experimental infections should be performed. These should involve detailed and multidisciplinary approaches in analysis of proteomes, secretomes and cestode exosomes in cysticercoid and other juvenile stages of the parasite.

## Additional files

Additional file 1: Table S1. Results of the LC-MS/MS analysis of selected spots. Proteins identified for cysticercoid Hymenolepis diminuta. **Table S2.** Functions of H. diminuta cysticercoid proteins according to their gene ontology (GO) categories. (DOCX 54 kb)

## Abbreviations

2DE: Two-dimensional electrophoresis
Apo-AI: Apolipoprotein AI
DTT: 1,4-Dithiothreitol
GO: Gene ontology
GRP78: 78 kDa glucose-regulated protein
HDL: High density lipoproteins
HSP: Heat shock protein
IEF: Isoelectric focusing
IPG: Immobilized pH gradient
LC-MS/MS: Liquid chromatography-mass spectrometry/mass spectrometry
MW: Molecular weight
PEPCK: Phosphoenolpyruvate carboxykinase
RBBP: Retinoblastoma binding protein
SDS-PAGE: Sodium dodecyl sulfate polyacrylamide gel electrophoresis
UPLC: Ultra performance liquid chromatography.
