# Supplementary material for: Identification of immunogenic proteins of the cysticercoid of Hymenolepis diminuta
Source: Parasit Vectors. 2017 Nov 21;10:577. doi: 10.1186/s13071-017-2519-4 (PMC5697066; doi:10.1186/s13071-017-2519-4)
Supplement: Supplementary file 1 — Results of the LC-MS/MS analysis of selected spots. Proteins identified for cysticercoid Hymenolepis diminuta. Table S2. Functions of H. diminuta cysticercoid proteins according to their gene ontology (GO) categories. (DOCX 54 kb) [file 13071_2017_2519_MOESM1_ESM.docx]

Additional file 1: Table S1. Results of the LC-MS/MS analysis of selected spots. Proteins identified for cysticercoid Hymenolepis diminuta.

| Spot | DB | Accession | Score | Mass | Matches | Sequences | emPAI | Sequence coverage (%) | Description |
| --- | --- | --- | --- | --- | --- | --- | --- | --- | --- |
| 1 | NCBInr | gi\|961498737 | 214 | 159681 | 5 | 3 | 0.11 | 1 | Type II collagen B [*Hymenolepis microstoma*] |
|  | NCBInr | gi\|576698379 | 183 | 127453 | 4 | 3 | 0.11 | 2 | Collagen alpha-1(XXVII) chain [*Echinococcus granulosus*] |
| 2 | NCBInr | gi\|961498737 | 150 | 159681 | 4 | 4 | 0.08 | 2 | Type II collagen B [*Hymenolepis microstoma*] |
|  | NCBInr | gi\|467215 | 116 | 41700 | 3 | 3 | 0.26 | 8 | Actin, partial [*Diphyllobothrium dendriticum*] |
|  | NCBInr | gi\|1703124 | 110 | 41896 | 3 | 3 | 0.26 | 7 | Actin-6 |
| 3 | NCBInr | gi\|961498738 | 285 | 135017 | 7 | 5 | 0.15 | 4 | Hypothetical transcript [*Hymenolepis microstoma*] |
| 4 | NCBInr | gi\|961498738 | 204 | 135017 | 5 | 5 | 0.13 | 4 | Hypothetical transcript [*Hymenolepis microstoma*] |
|  | NCBInr | gi\|961498737 | 101 | 159681 | 3 | 3 | 0.06 | 1 | Type II collagen B [*Hymenolepis microstoma*] |
| 5 | NCBInr | gi\|961498738 | 216 | 135017 | 5 | 4 | 0.10 | 2 | Hypothetical transcript [*Hymenolepis microstoma*] |
| 6 | NCBInr | gi\|576698378 | 176 | 130307 | 4 | 3 | 0.08 | 2 | Collagen alpha-1(XXVII) chain [*Echinococcus granulosus*] |
|  | NCBInr | gi\|961498737 | 109 | 159681 | 3 | 3 | 0.06 | 1 | Type II collagen B [*Hymenolepis microstoma*] |
| 7 | NCBInr | gi\|576698378 | 107 | 130307 | 3 | 2 | 0.05 | 1 | Collagen alpha-1(XXVII) chain [*Echinococcus granulosus*] |
| 8 | NCBInr | gi\|576693212 | 127 | 42176 | 4 | 4 | 0.35 | 10 | Actin, cytoplasmic 2 [*Echinococcus granulosus*] |
|  | NCBInr | gi\|961498737 | 108 | 159681 | 3 | 3 | 0.06 | 1 | Type II collagen B [*Hymenolepis microstoma*] |
| 9 | NCBInr | gi\|961497253 | 143 | 178165 | 3 | 3 | 0.06 | 2 | Collagen alpha 1(V) chain [*Hymenolepis microstoma*] |
| 10 | NCBInr | gi\|576693212 | 306 | 42176 | 6 | 5 | 0.57 | 13 | Actin, cytoplasmic 2 [*Echinococcus granulosus*] |
|  | NCBInr | gi\|1703110 | 231 | 42031 | 5 | 5 | 0.46 | 13 | Actin-2 |
|  | NCBInr | gi\|1703124 | 137 | 41896 | 4 | 4 | 0.36 | 9 | Actin-6 |
|  | NCBInr | gi\|674563099 | 109 | 123871 | 2 | 2 | 0.05 | 1 | Collagen type i ii iii v xi alpha [*Echinococcus granulosus*] |
| 11 | NCBInr | gi\|467215 | 293 | 41700 | 7 | 7 | 0.70 | 18 | Actin, partial [*Diphyllobothrium dendriticum*] |
|  | NCBInr | gi\|576693212 | 262 | 42176 | 6 | 6 | 0.57 | 14 | Actin, cytoplasmic 2 [*Echinococcus granulosus*] |
| 12 | NCBInr | gi\|674587834 | 284 | 224303 | 6 | 6 | 0.09 | 3 | Myosin heavy chain [*Hymenolepis microstoma*] |
|  | NCBInr | gi\|576698220 | 280 | 227397 | 6 | 6 | 0.09 | 3 | Myosin heavy chain, striated muscle [*Echinococcus granulosus*] |
|  | NCBInr | gi\|674563099 | 113 | 123871 | 2 | 2 | 0.05 | 1 | Collagen type i ii iii v xi alpha [*Echinococcus granulosus*] |
| 13 | NCBInr | gi\|674563099 | 108 | 123871 | 2 | 2 | 0.05 | 1 | Collagen type i ii iii v xi alpha [*Echinococcus granulosus*] |
| 14 | NCBInr | gi\|961497681 | 127 | 232229 | 3 | 3 | 0.04 | 1 | Myosin heavy chain non muscle [*Hymenolepis microstoma*] |
| 15 | NCBInr | gi\|961497252 | 119 | 38082 | 3 | 2 | 0.28 | 4 | Collagen alpha 1(V) chain, partial [*Hymenolepis microstoma*] |
|  | NCBInr | gi\|674563099 | 113 | 123871 | 2 | 2 | 0.05 | 1 | Collagen type i ii iii v xi alpha [*Echinococcus* *granulosus*] |
|  | NCBInr | gi\|961496216 | 112 | 126008 | 3 | 2 | 0.05 | 1 | Collagen alpha 2(I) chain [*Hymenolepis* *microstoma*] |
| 16 | NCBInr | gi\|961498737 | 457 | 159681 | 11 | 6 | 0.20 | 3 | Type II collagen B [*Hymenolepis* *microstoma*] |
| 17 | NCBInr | gi\|674572459 | 574 | 155578 | 12 | 8 | 0.23 | 3 | Type II collagen B [*Echinococcus* *multilocularis*] |
|  | NCBInr | gi\|961497253 | 117 | 178165 | 3 | 3 | 0.06 | 1 | Collagen alpha 1(V) chain [*Hymenolepis* *microstoma*] |
| 18 | NCBInr | gi\|674572459 | 532 | 155578 | 10 | 7 | 0.18 | 3 | Type II collagen B [*Echinococcus* *multilocularis*] |
| 19 | NCBInr | gi\|674572459 | 535 | 155578 | 11 | 8 | 0.21 | 3 | Type II collagen B [*Echinococcus* *multilocularis*] |
| 20 | NCBInr | gi\|674572459 | 343 | 155578 | 8 | 7 | 0.18 | 3 | Type II collagen B [*Echinococcus* *multilocularis*] |
|  | NCBInr | gi\|576693212 | 145 | 42176 | 4 | 4 | 0.35 | 11 | Actin, cytoplasmic 2 [*Echinococcus* *granulosus*] |
| 21 | NCBInr | gi\|674572459 | 298 | 155578 | 7 | 6 | 0.16 | 2 | Type II collagen B [*Echinococcus* *multilocularis*] |
| 22 | NCBInr | gi\|674572459 | 299 | 155578 | 7 | 5 | 0.13 | 2 | Type II collagen B [*Echinococcus* *multilocularis*] |
|  | NCBInr | gi\|674591691 | 120 | 36189 | 3 | 3 | 0.30 | 7 | ATP dependent RNA helicase Ddx1 [*Hymenolepis* *microstoma*] |
| 23 | NCBInr | gi\|576698379 | 309 | 127453 | 7 | 5 | 0.16 | 2 | Collagen alpha-1(XXVII) chain [*Echinococcus* *granulosus*] |
|  | NCBInr | gi\|961498737 | 274 | 159681 | 6 | 4 | 0.11 | 1 | Type II collagen B [*Hymenolepis* *microstoma*] |
| 24 | NCBInr | gi\|674572459 | 223 | 155578 | 5 | 4 | 0.11 | 1 | Type II collagen B [*Echinococcus* *multilocularis*] |
|  | NCBInr | gi\|576693212 | 138 | 42176 | 4 | 4 | 0.35 | 10 | Actin, cytoplasmic 2 [*Echinococcus* *granulosus*] |
|  | NCBInr | gi\|674595722 | 119 | 82804 | 3 | 3 | 0.12 | 3 | Procollagen lysine2 oxoglutarate 5 dioxygenase [*Hymenolepis* *microstoma*] |
| 25 | NCBInr | gi\|674595721 | 160 | 80042 | 5 | 4 | 0.22 | 5 | Procollagen lysine 2 oxoglutarate 5 dioxygenase [*Hymenolepis* *microstoma*] |
|  | NCBInr | gi\|576696179 | 101 | 100682 | 4 | 2 | 0.10 | 1 | Transforming growth factor-beta-induced protein ig-h3 [*Echinococcus* *granulosus*] |
|  | NCBInr | gi\|674591400 | 75 | 101853 | 3 | 3 | 0.10 | 2 | Lysosomal alpha glucosidase [*Hymenolepis* *microstoma*] |
| 26 | NCBInr | gi\|674595721 | 174 | 80042 | 5 | 4 | 0.22 | 5 | Procollagen lysine 2 oxoglutarate 5 dioxygenase [*Hymenolepis* *microstoma*] |
|  | NCBInr | gi\|674587303 | 119 | 87425 | 4 | 3 | 0.16 | 3 | Gynecophoral canal protein [*Hymenolepis* *microstoma*] |
|  | NCBInr | gi\|576696179 | 106 | 100682 | 4 | 3 | 0.14 | 2 | Transforming growth factor-beta-induced protein ig-h3 [*Echinococcus* *granulosus*] |
|  | NCBInr | gi\|1703124 | 102 | 41896 | 3 | 3 | 0.26 | 6 | Actin-6 |
| 27 | NCBInr | gi\|576693212 | 192 | 42176 | 5 | 5 | 0.46 | 13 | Actin, cytoplasmic 2 [*Echinococcus* *granulosus*] |
|  | NCBInr | gi\|674595721 | 183 | 80042 | 6 | 4 | 0.27 | 5 | Procollagen lysine 2 oxoglutarate 5 dioxygenase [*Hymenolepis* *microstoma*] |
|  | NCBInr | gi\|674587303 | 161 | 87425 | 4 | 3 | 0.16 | 3 | Gynecophoral canal protein [*Hymenolepis* *microstoma*] |
|  | NCBInr | gi\|576696179 | 119 | 100682 | 4 | 3 | 0.14 | 2 | Transforming growth factor-beta-induced protein ig-h3 [*Echinococcus* *granulosus*] |
|  | NCBInr | gi\|961498176 | 136 | 271951 | 4 | 4 | 0.05 | 1 | Spectrin beta chain [*Hymenolepis* *microstoma*] |
| 28 | NCBInr | gi\|674595721 | 209 | 80042 | 7 | 5 | 0.32 | 6 | Procollagen lysine 2 oxoglutarate 5 dioxygenase [*Hymenolepis* *microstoma*] |
| 29 | NCBInr | gi\|674595337 | 169 | 354920 | 4 | 4 | 0.04 | 1 | Nuclear pore complex protein Nup205 [*Hymenolepis* *microstoma*] |
|  | NCBInr | gi\|674591003 | 162 | 66019 | 5 | 5 | 0.27 | 6 | Heat shock protein 70 [*Hymenolepis* *microstoma*] |
|  | NCBInr | gi\|1661112 | 133 | 70925 | 3 | 3 | 0.15 | 4 | Heat shock 70kDa protein, partial [*Mesocestoides* *corti*] |
|  | NCBInr | gi\|576696491 | 116 | 78208 | 4 | 4 | 0.18 | 4 | Stress-70 protein [*Echinococcus* *granulosus*] |
|  | NCBInr | gi\|674595722 | 130 | 82804 | 3 | 3 | 0.12 | 3 | Procollagen lysine2 oxoglutarate 5 dioxygenase [*Hymenolepis* *microstoma*] |
|  | NCBInr | gi\|576693212 | 129 | 42176 | 3 | 3 | 0.25 | 8 | Actin, cytoplasmic 2 [*Echinococcus* *granulosus*] |
|  | NCBInr | gi\|674593372 | 106 | 71158 | 3 | 3 | 0.14 | 4 | Phosphoenolpyruvate carboxykinase [*Hymenolepis* *microstoma*] |
| 30 | NCBInr | gi\|961498829 | 330 | 70864 | 5 | 5 | 0.25 | 8 | Hypothetical transcript [*Hymenolepis* *microstoma*] |
|  | NCBInr | gi\|674591003 | 325 | 66019 | 8 | 8 | 0.47 | 12 | Heat shock protein 70 [*Hymenolepis* *microstoma*] |
|  | NCBInr | gi\|1661112 | 255 | 70925 | 5 | 5 | 0.25 | 7 | Heat shock 70kDa protein, partial [*Mesocestoides* *corti*] |
|  | NCBInr | gi\|576696491 | 223 | 78208 | 6 | 6 | 0.28 | 7 | Stress-70 protein [*Echinococcus* *granulosus*] |
|  | NCBInr | gi\|674587174 | 203 | 274858 | 3 | 3 | 0.04 | 1 | Filamin [*Hymenolepis* *microstoma*] |
|  | NCBInr | gi\|674595721 | 168 | 80042 | 5 | 4 | 0.22 | 5 | Procollagen lysine 2 oxoglutarate 5 dioxygenase [*Hymenolepis* *microstoma*] |
|  | NCBInr | gi\|674589006 | 160 | 85044 | 4 | 4 | 0.16 | 4 | Hypothetical transcript [*Hymenolepis* *microstoma*] |
|  | NCBInr | gi\|576693212 | 159 | 42176 | 4 | 4 | 0.35 | 10 | Actin, cytoplasmic 2 [*Echinococcus* *granulosus*] |
|  | NCBInr | gi\|674586556 | 113 | 88988 | 3 | 3 | 0.11 | 3 | Calpain A [*Hymenolepis* *microstoma*] |
|  | NCBInr | gi\|674593372 | 108 | 71158 | 3 | 3 | 0.14 | 4 | Phosphoenolpyruvate carboxykinase [*Hymenolepis* *microstoma*] |
| 31 | NCBInr | gi\|576693212 | 215 | 42176 | 6 | 4 | 0.57 | 10 | Actin, cytoplasmic 2 [*Echinococcus* *granulosus*] |
|  | NCBInr | gi\|1703124 | 121 | 41896 | 4 | 3 | 0.36 | 6 | Actin-6 |
|  | NCBInr | gi\|674589006 | 162 | 85044 | 4 | 4 | 0.16 | 4 | Hypothetical transcript [*Hymenolepis* *microstoma*] |
|  | NCBInr | gi\|674595722 | 149 | 82804 | 3 | 3 | 0.12 | 3 | Procollagen lysine2 oxoglutarate 5 dioxygenase [*Hymenolepis* *microstoma*] |
| 32 | NCBInr | gi\|674593792 | 664 | 72678 | 16 | 12 | 0.86 | 14 | Heat Shock protein family member (hsp 3) [*Hymenolepis* *microstoma*] |
|  | NCBInr | gi\|29336626 | 591 | 71859 | 15 | 12 | 0.87 | 14 | 78 kDa glucose-regulated protein; Short=GRP-78; Flags: Precursor |
|  | NCBInr | gi\|32481989 | 511 | 71597 | 10 | 7 | 0.50 | 10 | Glucose regulated protein GRP78 [*Spirometra* *erinaceieuropaei*] |
|  | NCBInr | gi\|961497969 | 254 | 100726 | 6 | 6 | 0.21 | 6 | Paramyosin [*Hymenolepis* *microstoma*] |
|  | NCBInr | gi\|961497681 | 249 | 232229 | 7 | 7 | 0.10 | 2 | Myosin heavy chain non muscle [*Hymenolepis* *microstoma*] |
|  | NCBInr | gi\|576700458 | 139 | 230063 | 4 | 4 | 0.06 | 1 | Myosin-11 [*Echinococcus* *granulosus*] |
|  | NCBInr | gi\|674587834 | 246 | 224303 | 5 | 5 | 0.07 | 2 | Myosin heavy chain [*Hymenolepis* *microstoma*] |
|  | NCBInr | gi\|576698220 | 134 | 227397 | 5 | 5 | 0.07 | 2 | Myosin heavy chain, striated muscle [*Echinococcus* *granulosus*] |
|  | NCBInr | gi\|674587174 | 160 | 274858 | 4 | 4 | 0.05 | 1 | Filamin [*Hymenolepis* *microstoma*] |
|  | NCBInr | gi\|576696380 | 157 | 282724 | 4 | 4 | 0.05 | 1 | Spectrin alpha chain [*Echinococcus* *granulosus*] |
| 33 | NCBInr | gi\|674593792 | 526 | 72678 | 12 | 9 | 0.56 | 11 | Heat Shock protein family member (hsp 3) [*Hymenolepis* *microstoma*] |
|  | NCBInr | gi\|32481989 | 360 | 71597 | 6 | 5 | 0.31 | 9 | Glucose regulated protein GRP78 [*Spirometra* *erinaceieuropaei*] |
|  | NCBInr | gi\|674587174 | 442 | 274858 | 10 | 8 | 0.13 | 3 | Filamin [*Hymenolepis* *microstoma*] |
|  | NCBInr | gi\|961497681 | 303 | 232229 | 8 | 7 | 0.12 | 3 | Myosin heavy chain non muscle [*Hymenolepis* *microstoma*] |
|  | NCBInr | gi\|576700458 | 204 | 230063 | 5 | 5 | 0.07 | 2 | Myosin-11 [*Echinococcus* *granulosus*] |
|  | NCBInr | gi\|576696380 | 227 | 282724 | 7 | 7 | 0.08 | 2 | Spectrin alpha chain [*Echinococcus* *granulosus*] |
|  | NCBInr | gi\|674587834 | 165 | 224303 | 4 | 4 | 0.06 | 2 | Myosin heavy chain [*Hymenolepis* *microstoma*] |
|  | NCBInr | gi\|674572459 | 140 | 155578 | 3 | 3 | 0.06 | 1 | Type II collagen B [*Echinococcus* *multilocularis*] |
| 34 | NCBInr | gi\|576701165 | 665 | 57142 | 15 | 12 | 1.32 | 27 | Tubulin alpha-1C chain [*Echinococcus* *granulosus*] |
|  | NCBInr | gi\|674580112 | 656 | 60984 | 12 | 11 | 0.88 | 22 | Heat shock protein 60 [*Echinococcus* *multilocularis*] |
|  | NCBInr | gi\|961498402 | 567 | 61181 | 11 | 10 | 0.78 | 19 | Hypothetical transcript [*Hymenolepis* *microstoma*] |
|  | NCBInr | gi\|674587834 | 343 | 224303 | 6 | 6 | 0.09 | 3 | Myosin heavy chain [*Hymenolepis* *microstoma*] |
|  | NCBInr | gi\|961497681 | 339 | 232229 | 8 | 7 | 0.12 | 3 | Myosin heavy chain non muscle [*Hymenolepis* *microstoma*] |
|  | NCBInr | gi\|576700458 | 214 | 230063 | 4 | 4 | 0.06 | 2 | Myosin-11 [*Echinococcus* *granulosus*] |
|  | NCBInr | gi\|674591524 | 319 | 282888 | 8 | 7 | 0.10 | 3 | Spectrin alpha actinin [*Hymenolepis* *microstoma*] |
|  | NCBInr | gi\|674586556 | 160 | 88988 | 4 | 4 | 0.16 | 3 | Calpain A [*Hymenolepis* *microstoma*] |
| 35 | NCBInr | gi\|674266667 | 197 | 282599 | 6 | 6 | 0.07 | 2 | Spectrin alpha actinin [*Echinococcus* *multilocularis*] |
|  | NCBInr | gi\|674572459 | 174 | 155578 | 4 | 3 | 0.09 | 1 | Type II collagen B [*Echinococcus* *multilocularis*] |
|  | NCBInr | gi\|576693212 | 132 | 42176 | 4 | 4 | 0.35 | 11 | Actin, cytoplasmic 2 [*Echinococcus* *granulosus*] |
|  | NCBInr | gi\|961498402 | 131 | 61181 | 4 | 4 | 0.23 | 6 | Hypothetical transcript [*Hymenolepis* *microstoma*] |
|  | NCBInr | gi\|674587834 | 126 | 224303 | 5 | 5 | 0.07 | 2 | Myosin heavy chain [*Hymenolepis* *microstoma*] |
|  | NCBInr | gi\|576698220 | 116 | 227397 | 5 | 5 | 0.07 | 2 | Myosin heavy chain, striated muscle [*Echinococcus* *granulosus*] |
|  | NCBInr | gi\|674586556 | 126 | 88988 | 3 | 3 | 0.11 | 2 | Calpain A [*Hymenolepis microstoma*] |
|  | NCBInr | gi\|674595558 | 121 | 61301 | 4 | 4 | 0.23 | 6 | Dihydropyrimidinase 2 [*Hymenolepis microstoma*] |
|  | NCBInr | gi\|421975927 | 120 | 50838 | 3 | 3 | 0.21 | 5 | Tubulin [*Spirometra erinaceieuropaei*] |
|  | NCBInr | gi\|576692751 | 117 | 163172 | 3 | 3 | 0.06 | 1 | Tubulin alpha-1C chain [*Echinococcus granulosus*] |
|  | NCBInr | gi\|576700458 | 67 | 230063 | 3 | 3 | 0.04 | 1 | Myosin-11 [*Echinococcus granulosus*] |
| 36 | NCBInr | gi\|961498429 | 363 | 320791 | 7 | 7 | 0.07 | 2 | Apolipoprotein A I binding protein [*Hymenolepis microstoma*] |
|  | NCBInr | gi\|576695222 | 357 | 317954 | 8 | 7 | 0.08 | 2 | Talin-1 [*Echinococcus granulosus*] |
|  | NCBInr | gi\|961497045 | 201 | 89288 | 6 | 6 | 0.24 | 6 | Radixin [*Hymenolepis microstoma*] |
|  | NCBInr | gi\|674586556 | 135 | 88988 | 3 | 3 | 0.11 | 3 | Calpain A [*Hymenolepis microstoma*] |
|  | NCBInr | gi\|674593372 | 90 | 71158 | 3 | 3 | 0.14 | 4 | Phosphoenolpyruvate carboxykinase [*Hymenolepis microstoma*] |
| 37 | NCBInr | gi\|674595068 | 720 | 50360 | 18 | 14 | 1.59 | 33 | Tubulin beta 2C chain [*Hymenolepis microstoma*] |
|  | NCBInr | gi\|674589522 | 398 | 50888 | 10 | 9 | 0.87 | 19 | Tubulin beta 1 chain [*Hymenolepis microstoma*] |
|  | NCBInr | gi\|674589523 | 341 | 50361 | 9 | 8 | 0.77 | 17 | Beta tubulin [*Hymenolepis microstoma*] |
|  | NCBInr | gi\|237770557 | 229 | 15158 | 6 | 4 | 1.74 | 29 | Beta-tubulin isoform 2, partial [*Echinococcus granulosus*] |
|  | NCBInr | gi\|674586556 | 187 | 88988 | 5 | 5 | 0.20 | 4 | Calpain A [*Hymenolepis microstoma*] |
|  | NCBInr | gi\|674591321 | 167 | 49186 | 4 | 4 | 0.30 | 8 | Retinoblastoma binding protein 4 [*Hymenolepis microstoma*] |
|  | NCBInr | gi\|467215 | 122 | 41700 | 3 | 3 | 0.26 | 8 | Actin, partial [*Diphyllobothrium dendriticum*] |
| 38 | NCBInr | gi\|961500922 | 799 | 38431 | 17 | 12 | 2.75 | 37 | Hypothetical transcript [*Hymenolepis microstoma*] |
|  | NCBInr | gi\|674595068 | 672 | 50360 | 19 | 13 | 1.43 | 28 | Tubulin beta 2C chain [*Hymenolepis microstoma*] |
|  | NCBInr | gi\|29337144 | 636 | 50269 | 18 | 13 | 1.28 | 28 | Tubulin beta-2 chain |
|  | NCBInr | gi\|347309784 | 314 | 41990 | 10 | 5 | 0.46 | 13 | Beta-tubulin, partial [*Hymenolepis* *microstoma*] |
|  | NCBInr | gi\|674593099 | 145 | 52345 | 4 | 4 | 0.28 | 6 | Beta tubulin [*Hymenolepis microstoma*] |
|  | NCBInr | gi\|674592610 | 302 | 48894 | 7 | 7 | 0.58 | 14 | ATP dependent RNA helicase DDX31 [*Hymenolepis microstoma*] |
|  | NCBInr | gi\|576697500 | 296 | 48796 | 7 | 7 | 0.58 | 14 | 26S protease regulatory subunit 7 [*Echinococcus granulosus*] |
|  | NCBInr | gi\|576698586 | 208 | 61520 | 5 | 5 | 0.30 | 8 | 26S protease regulatory subunit 6A [*Echinococcus granulosus*] |
|  | NCBInr | gi\|29336626 | 251 | 71859 | 6 | 6 | 0.31 | 9 | 78 kDa glucose-regulated protein |
|  | NCBInr | gi\|1661112 | 130 | 70925 | 3 | 3 | 0.15 | 5 | Heat shock 70kDa protein, partial [*Mesocestoides corti*] |
|  | NCBInr | gi\|674588688 | 251 | 53767 | 6 | 5 | 0.43 | 9 | Mitochondrial processing peptidase beta subunit [*Hymenolepis microstoma*] |
|  | NCBInr | gi\|576693212 | 220 | 42176 | 5 | 5 | 0.46 | 13 | Actin, cytoplasmic 2 [*Echinococcus granulosus*] |
|  | NCBInr | gi\|961499185 | 188 | 42368 | 3 | 3 | 0.25 | 7 | Succinate coenzyme A ligase, GDP forming, beta subunit [*Hymenolepis microstoma*] |
|  | NCBInr | gi\|576693631 | 169 | 49556 | 3 | 3 | 0.21 | 6 | Succinyl-CoA ligase [GDP-forming] subunit beta [*Echinococcus granulosus*] |
|  | NCBInr | gi\|6467323 | 141 | 29776 | 4 | 4 | 0.53 | 12 | Elongation factor 1-a, partial [*Hymenolepis diminuta*] |
|  | NCBInr | gi\|674595901 | 115 | 49810 | 4 | 4 | 0.29 | 7 | Lamin dm0 [*Hymenolepis microstoma*] |
|  | NCBInr | gi\|674585637 | 107 | 63550 | 3 | 3 | 0.16 | 4 | NADP dependent malic enzyme [*Hymenolepis microstoma*] |
|  | NCBInr | gi\|674587985 | 87 | 48883 | 3 | 3 | 0.22 | 6 | Dihydrolipoyllysine residue succinyltransferase [*Hymenolepis microstoma*] |
| 39 | NCBInr | gi\|674589423 | 342 | 35298 | 10 | 5 | 0.87 | 13 | Major egg antigen (p40) [*Hymenolepis microstoma*] |
|  | NCBInr | gi\|17065922 | 313 | 35690 | 9 | 5 | 0.86 | 14 | Putative HSP20 related protein [*Echinococcus multilocularis*] |
|  | NCBInr | gi\|21665905 | 172 | 35640 | 5 | 3 | 0.31 | 10 | Small heat-shock protein [*Taenia solium*] |
|  | NCBInr | gi\|674587174 | 264 | 274858 | 4 | 4 | 0.05 | 1 | Filamin [*Hymenolepis microstoma*] |
|  | NCBInr | gi\|674595570 | 238 | 41700 | 6 | 6 | 0.58 | 14 | Stomatin protein 2 [*Hymenolepis microstoma*] |
|  | NCBInr | gi\|961500922 | 235 | 38431 | 5 | 4 | 0.39 | 11 | Hypothetical transcript [*Hymenolepis microstoma*] |
|  | NCBInr | gi\|576693212 | 218 | 42176 | 5 | 5 | 0.46 | 12 | Actin, cytoplasmic 2 [*Echinococcus granulosus*] |
|  | NCBInr | gi\|1703110 | 214 | 42031 | 5 | 5 | 0.46 | 12 | Actin-2 |
|  | NCBInr | gi\|674595068 | 210 | 50360 | 4 | 4 | 0.29 | 9 | Tubulin beta 2C chain [*Hymenolepis microstoma*] |
|  | NCBInr | gi\|674589522 | 172 | 50888 | 4 | 4 | 0.29 | 9 | Tubulin beta 1 chain [*Hymenolepis microstoma*] |
|  | NCBInr | gi\|29337143 | 161 | 50252 | 4 | 4 | 0.29 | 9 | Tubulin beta-3 chain |
|  | NCBInr | gi\|961498852 | 161 | 53277 | 5 | 5 | 0.35 | 9 | Chaperonin containing TCP1 subunit 5 (epsilon) [*Hymenolepis microstoma*] |
|  | NCBInr | gi\|674593856 | 154 | 57534 | 3 | 3 | 0.18 | 6 | Chaperonin containing TCP1 subunit 2 (beta) [*Hymenolepis microstoma*] |
|  | NCBInr | gi\|674587773 | 142 | 308234 | 4 | 4 | 0.04 | 1 | Filamin [*Hymenolepis microstoma*] |
|  | NCBInr | gi\|576698586 | 119 | 61520 | 3 | 3 | 0.17 | 4 | 26S protease regulatory subunit 6A [*Echinococcus granulosus*] |
| 40 | NCBInr | gi\|576693212 | 2595 | 42176 | 65 | 16 | 5.11 | 40 | Actin, cytoplasmic 2 [*Echinococcus granulosus*] |
|  | NCBInr | gi\|1703110 | 2577 | 42031 | 62 | 15 | 5.14 | 40 | Actin-2 |
|  | NCBInr | gi\|1703122 | 2360 | 41442 | 59 | 15 | 4.42 | 37 | Actin-5 |
|  | NCBInr | gi\|543766 | 730 | 42143 | 14 | 7 | 0.83 | 21 | Actin-1 |
|  | NCBInr | gi\|674589522 | 182 | 50888 | 3 | 3 | 0.21 | 7 | Tubulin beta 1 chain [*Hymenolepis microstoma*] |
|  | NCBInr | gi\|29337144 | 105 | 50269 | 3 | 3 | 0.21 | 7 | Tubulin beta-2 chain |
|  | NCBInr | gi\|961500921 | 136 | 42989 | 3 | 3 | 0.25 | 7 | Hypothetical transcript [*Hymenolepis microstoma*] |
| 41 | NCBInr | gi\|576693212 | 2115 | 42176 | 43 | 17 | 7.91 | 41 | Actin, cytoplasmic 2 [*Echinococcus granulosus*] |
|  | NCBInr | gi\|1703110 | 2114 | 42031 | 42 | 16 | 7.96 | 41 | Actin-2 |
|  | NCBInr | gi\|1703122 | 1950 | 41442 | 41 | 16 | 7.59 | 39 | Actin-5 |
|  | NCBInr | gi\|543766 | 771 | 42143 | 15 | 9 | 1.67 | 25 | Actin-1 |
|  | NCBInr | gi\|576696380 | 214 | 282724 | 5 | 5 | 0.06 | 2 | Spectrin alpha chain [*Echinococcus granulosus*] |
|  | NCBInr | gi\|442093123 | 200 | 56214 | 4 | 4 | 0.26 | 8 | Mitochondrial ATP synthase [*Spirometra erinaceieuropaei*] |
|  | NCBInr | gi\|576692679 | 188 | 71324 | 6 | 6 | 0.31 | 8 | Heat shock cognate protein [*Echinococcus granulosus*] |
|  | NCBInr | gi\|961498830 | 179 | 41026 | 6 | 6 | 0.59 | 13 | Hypothetical transcript [*Hymenolepis microstoma*] |
|  | NCBInr | gi\|1661112 | 171 | 70925 | 6 | 6 | 0.31 | 8 | Heat shock 70kDa protein, partial [*Mesocestoides corti*] |
|  | NCBInr | gi\|674589523 | 188 | 50361 | 4 | 4 | 0.29 | 9 | Beta tubulin [*Hymenolepis microstoma*] |
|  | NCBInr | gi\|674589522 | 157 | 50888 | 3 | 3 | 0.21 | 7 | Tubulin beta 1 chain [*Hymenolepis microstoma*] |
|  | NCBInr | gi\|674592610 | 168 | 48894 | 5 | 5 | 0.39 | 10 | ATP dependent RNA helicase DDX31 [*Hymenolepis microstoma*] |
|  | NCBInr | gi\|961500921 | 151 | 42989 | 4 | 4 | 0.35 | 9 | Hypothetical transcript [*Hymenolepis microstoma*] |
| 42 | NCBInr | gi\|29337144 | 366 | 50269 | 9 | 8 | 0.66 | 17 | Tubulin beta-2 chain |
|  | NCBInr | gi\|29336623 | 339 | 72851 | 7 | 6 | 0.36 | 12 | Heat shock cognate 70 kDa protein |
|  | NCBInr | gi\|32481989 | 164 | 71597 | 4 | 4 | 0.20 | 9 | Glucose regulated protein GRP78 [*Spirometra erinaceieuropaei*] |
|  | NCBInr | gi\|961496319 | 184 | 26510 | 3 | 3 | 0.43 | 12 | Hypothetical transcript [*Hymenolepis microstoma*] |
|  | NCBInr | gi\|576697794 | 178 | 62384 | 3 | 3 | 0.17 | 5 | Putative cyclin-H [*Echinococcus granulosus*] |
|  | NCBInr | gi\|467215 | 113 | 41700 | 3 | 3 | 0.26 | 7 | Actin, partial [*Diphyllobothrium dendriticum*] |
|  | NCBInr | gi\|576699883 | 76 | 38846 | 3 | 3 | 0.28 | 8 | Annexin A7 [*Echinococcus granulosus*] |

Additional file 1: Table S2. Functions of H. diminuta cysticercoid proteins according to their gene ontology (GO) categories.

| Subcategory | Gen ontology numer (GO) | Number of proteins |
| --- | --- | --- |
|  |  |  |
| Molecular function (GO: 0003674) | | |
| phosphoenolpyruvate carboxykinase (GTP) activity | GO:0004613 | 1 |
| oxidoreductase activity | GO:0016491 | 3 |
| transferase activity | GO:0016740 | 3 |
| hydrolase activity | GO:0016787 | 24 |
| ligase activity | GO: 0016874 | 2 |
| structural molecule activity | GO:0005198 | 16 |
| transporter activity | GO:0005215 | 1 |
| phospholipid binding | GO:0005543 | 2 |
| carbohydrate binding | GO:0030246 | 1 |
| small molecule binding | GO:0036094 | 40 |
| ion binding | GO:0043167 | 6 |
| NAD binding | GO:0051287 | 1 |
| iron sulfur cluster binding | GO:0051536 | 1 |
| organic cyclic compound binding | GO:0097159 | 39 |
| carbohydrate derivative binding | GO:0097367 | 38 |
| heterocyclic compound binding | GO:1901363 | 39 |
| electron carrier activity | GO:0009055 | 1 |
| protein phosphatase type 2A regulator activity |  | 1 |
| Biological process (GO:0008150) | | |
| cell adhesion | GO:0007155 | 2 |
| nitrogen compound metabolic process | GO:0006807 | 3 |
| catabolic process | GO:0009056 | 4 |
| biosynthetic process | GO:0009058 | 3 |
| cellular metabolic process | GO:0044237 | 7 |
| primary metabolic process | GO:0044238 | 12 |
| single-organism metabolic process | GO:0044710 | 6 |
| organic substance metabolic process | GO:0071704 | 13 |
| cellular process | GO:0009987 | 24 |
| single-organism process | GO:0044699 | 16 |
| localization | GO:0051179 | 3 |
| biological regulation | GO:0065007 | 3 |
| cellular component organization or biogenesis | GO:0071840 | 12 |
| Cellular component (GO:0005575) | | |
| extracellular region |  | 6 |
| proteinaceous extracellular matrix | GO:0005578 | 5 |
| cell | GO:0005623 | 35 |
| endoplasmic reticulum lumen | GO:0005788 | 1 |
| membrane | GO:0016020 | 4 |
| cell junction | GO:0030054 | 2 |
| organelle | GO:0043226 | 26 |
| protein complex | GO:0043234 | 14 |
| organelle part | GO:0044422 | 17 |
| membrane part | GO:0044425 | 2 |
| cell part | GO:0044464 | 35 |
| polymeric cytoskeletal fiber | GO:0099513 | 9 |
